# Efficacy of *Lactobacillus*-supplemented triple therapy for *H*. *pylori* eradication: A meta-analysis of randomized controlled trials

**DOI:** 10.1371/journal.pone.0223309

**Published:** 2019-10-02

**Authors:** Mingyang Yu, Rongguang Zhang, Peng Ni, Shuaiyin Chen, Guangcai Duan

**Affiliations:** Department of Epidemiology, College of Public Health, Zhengzhou University, Zhengzhou, China; Instiuto Ramon y Cajal de Investigacion Sanitaria (IRYCIS), SPAIN

## Abstract

**Aim:**

To assess the effect of *Lactobacillus* supplementation on *Helicobacter pylori* eradication rates and side effects of the triple therapy.

**Methods:**

PubMed, Embase, Web of Science and Cochrane Library were searched for articles published up to July, 2019. Review Manager 5.3 and Stata 12.0 were used for statistical analyses.

**Results:**

The initial database search resulted in 852 articles. Through exclusion and screening, 11 randomized controlled trials involving a total of 724 patients were finally included in this meta-analysis. The *H*. *pylori* elimination rate in the *Lactobacillus* supplement group was significantly higher than that in the control group (RR 1.16, 95% CI 1.08–1.25, *P*<0.0001). Subgroup analysis showed that the eradication rates were significantly enhanced in both adults and children group, and no significant difference was detected between Asia and Europe group. In addition, sub-analysis based on duration of *Lactobacillus* supplementation showed the pooled RRs in the long-term and short-term groups were 1.17 (95%CI 1.06–1.30) and 1.16 (95% CI 1.04–1.30), respectively. Regarding the *Lactobacillus* strains, the pooled RR was 1.33 (95% CI 1.10–1.62) in the *L*. *casei* group, 1.18 (95% CI 1.03–1.34) in the *L*. *reuteri* group while 1.02 (95% CI 0.87–1.21) in the *Lactobacillus GG* group. As for the total side effects, *Lactobacillus* supplementation significantly reduced the incidence of taste disturbance (RR = 0.36, 95% CI 0.17–0.74, *P* = 0.005).

**Conclusions:**

*Lactobacillus* supplementation during the treatment of *Helicobacter pylori* infection can effectively improve the eradication rates, and reduce the incidence of therapy-related taste disturbance.

## Introduction

*Helicobacter pylori*, a microaerophilic gram-negative bacterium, has quickly gained widespread attention since its discovery. *H*. *pylori* infection is closely associated with the incidence of gastrointestinal diseases [1,2]. In some developing countries, the prevalence of *H*. *pylori* is about 80–90*%* [3]. *H*. *pylori* infection has become a notable worldwide health problem. In 2016, The Toronto Consensus Reports (2016) reported the role of *H*. *pylori* in pathogenesis of gastrointestinal diseases [4]. Traditional triple therapy includes the proton pump inhibitor (PPI), clarithromycin and amoxicillin. The eradication rate of the traditional triple therapy could be 90% in early 1990s [5]. However, the eradication rate markedly decreased in recent decades, due to the increasing antibiotic resistance. In some countries, eradication failure rates currently exceed 20%, and they might be even higher in the areas with high prevalence of resistant *H*. *pylori* strains. [6–7]. Additionally, the therapies based on long-term or repeated use of antibiotics have also encountered problems on other side effects like alteration of gut microbiota [8]. Thus, it is necessary to improve the therapies or adjunctive treatments.

As microorganisms in mutually beneficial relation to human, probiotics can provide benefit in regulating gastrointestinal flora [9]. Some reviews showed that probiotics might have antagonistic effect on *Helicobacter pylori* [10]. Recently, some researches have used multistrain probiotics supplementation therapy for *H*. *pylori* eradication [11,12]. However, it seems that not all kinds of probiotics are effective for *H*. *pylori* eradication [13]. An alternative solution is to choose a clearly defined strain of probiotics that has been confirmed effective in inhibiting *H*. *pylori* in clinical trials. Studies have shown that some *Lactobacillus* strains can exhibit anti-*H*. *pylori* effects *in vitro* or in animal models [14–18]. A study by García et al. showed that the co-existence of *Lactobacillus* and *H*. *pylori* was low in the gastric biopsies of symptomatic patients [19]. If the co-existence of both species is reestablished, the growth of *H*. *pylori* may be inhibited. A network meta-analysis by Shi et al. showed that *Lactobacillus* might be a better choice of probiotic strains for eradicating *H*. *pylori* [20]. However, trials based on the efficacy of *Lactobacillus* in *H*. *pylori* eradication therapy were dramatically different in quality. Meanwhile, studies on the efficacy of *Lactobacillus* supplementation to the standard triple therapy are scant and lead to controversial conclusions.

Therefore, this meta-analysis was conducted to analyze whether the *Lactobacillus* -supplemented standard triple therapy can significantly increase the *H*. *pylori* eradication rates and reduce incidences of the side effects, aiming to provide more reliable evidences for clinical decision.

## Materials and methods

The Cochrane Handbook for Systematic Reviews of Interventions [21] and the PRISMA statement for reporting systematic reviews and meta-analysis [22] were followed for our meta-analysis.

### Search strategy

PubMed, Web of Science, Embase and Cochrane Library were systematically searched for all the relevant studies with no language restrictions (up to July 2019). The following search terms were used: (probiotics or probiotic or yogurt or *Lactobacillus*) and (*H*. *pylori* or *Helicobacter pylori* or *Campylobacter pylori* or *C*. *pylori*). In addition, our searches were limited to RCTs and controlled clinical trials. Reference lists of identified articles were also manually searched for other relevant articles. The complete search strategy was included in S1 Table.

### Selection criteria

Two independent reviewers reviewed the results of initial searches. Studies that met the following conditions were included in our meta-analysis: (1) studies using adequate and clear randomization methods; (2) patients with *H*. *pylori* infection diagnosed by histology, culture, rapid urease test, urea breath test, or *H*. *pylori* stool antigen test; (3) studies comparing at least two treatment groups including (a) triple therapy (a proton pump inhibitor and two antibiotics) with placebo or not and (b) the same regimen plus *Lactobacillus*; (4) confirmation of eradication outcomes at least four weeks after eradication therapy; (5) the full text was available; (6) the data of the eradication rates and/or side effects were available.

The following studies were excluded from the analysis: (1) case reports, comments, letters or reviews; (2) use of agents other than *Lactobacillus* as the adjuvant therapy for *H*. *pylori* infection in the experimental group; (3) use of oral antibiotics and/or PPIs and/or H2-antagonists during the 2 weeks prior to ingestion of the study product; (4) not RCTs.

In case of duplicate reports, or studies clearly reported results from the same study population, the latest or most complete one was selected.

### Data extraction

Two reviewers used the standardized data abstraction sheets to extract the data independently. Disagreements were resolved by consulting another reviewer. The following information was extracted from each included study: the first author’s name, published year, location, baseline features of the involved patients, diagnostic methods of testing *H*. *pylori* infection before enrolling and after completing study, *H*. *pylori* eradication regimen, *Lactobacillus* regimen, follow-up time, eradication rate as the primary outcome, and side effect rate as the secondary outcome.

### Quality assessment

The Cochrane risk of bias assessment tool [21] was used to evaluate the quality of the included studies, which include the following seven parameters: random sequence generation, allocation concealment, blinding of participants and personnel, blinding of outcome assessment, incomplete outcome data, selective reporting, and other bias. Each study was divided into three levels (low risk, unclear risk, and high risk). Review Manager 5.3 statistical software was used to assess these studies, and the results of them were showed as risk of bias graph and risk of bias summary. In addition, the quality of evidence for key outcomes was assessed using GRADEpro GDT. The GRADE system classifies quality of evidence as high, moderate, low, or very low according to factors that include the study methodology, consistency and precision of the results, and directness of the evidence [23]. Two independent reviewers completed the entire quality assessment. Consensus on quality assessment was reached via discussion.

### Statistical analysis

This meta-analysis was conducted using Review Manager 5.3 and Stata 12.0. The eradication and side effects rates were analyzed based on intent-to-treat (ITT) principle, and the dichotomous outcomes were expressed as relative risk (RR) with 95% confidence interval (CI). *P* < 0.05 was considered as statistically significant. Heterogeneity among studies was evaluated via chi-square tests and the inconsistency statistic. It indicated that there was significant heterogeneity if *I*^*2*^ > 50% and/or *P* < 0.1. Besides, the levels of heterogeneity assessed by *I*^2^ were as follows: 0–25% meant homogeneity; 25–50% meant low heterogeneity; 50–75% meant moderate heterogeneity, and >75% meant high heterogeneity [24]. The fixed-effects model (Mantel-Haensze) was used to analyze the data if no heterogeneity was present, while the random-effects model was used if *I*^2^ > 50% [25]. Subgroup analyses were conducted based on the ages, trial locations, duration, dose and species of *Lactobacillus* supplementation. In addition, Begg’s and Egger’s tests were used to detect potential publication bias. *P* values < 0.05 were considered as statistically significant.

## Results

### Literature search and study characteristics

**Fig 1:**
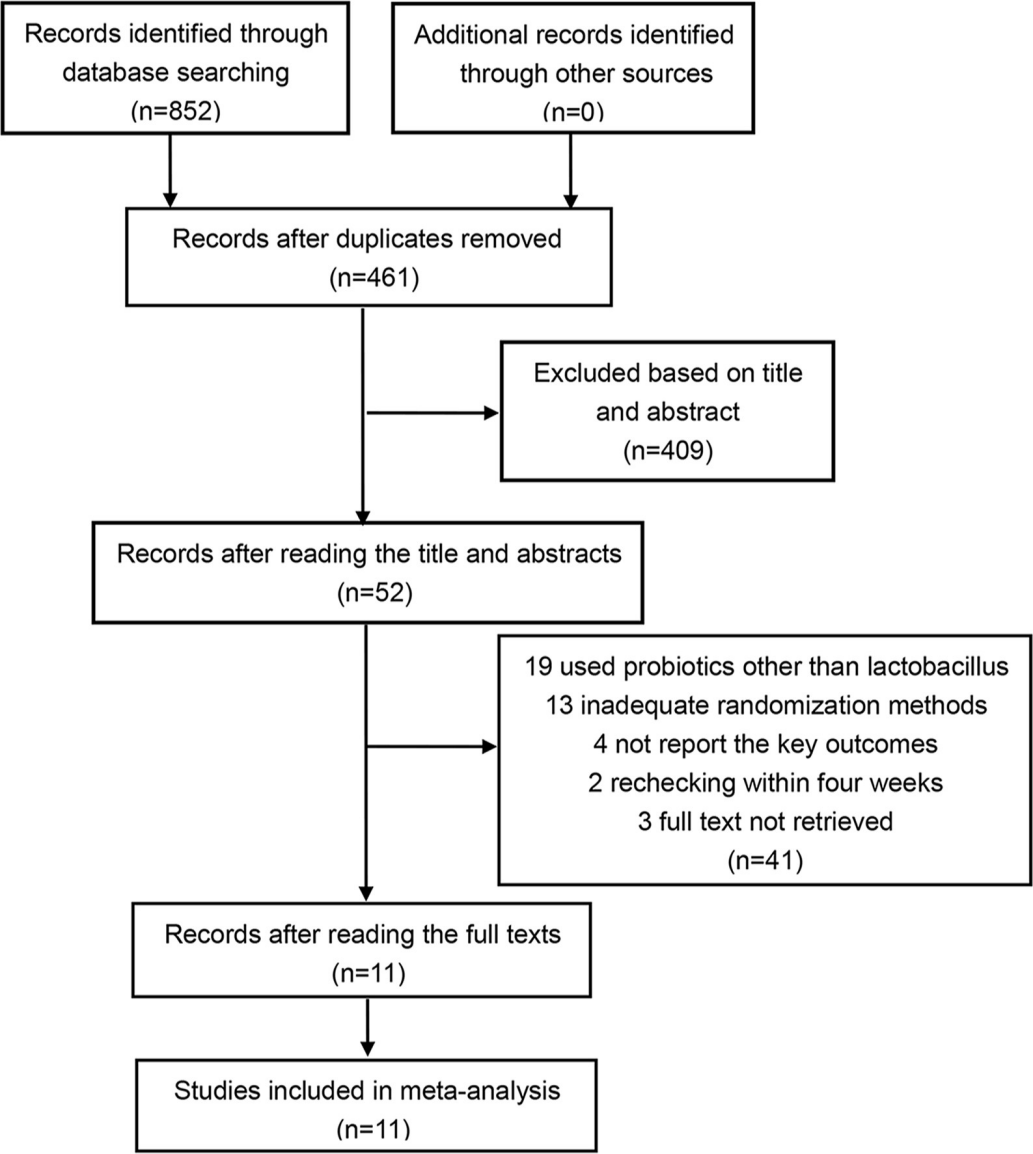
Flowchart of the study selection.

The initial database search generated 852 articles, 391 of which were excluded because of duplicates. After reading the title and abstract, 409 records were excluded via the initial screening. The whole 52 articles were screened and finally 11 studies were included for analysis [26–36]. Fig 1 details the selection and exclusion process.

The 11 RCTs included in this meta-analysis consisted of 972 patients. The publication years of the involved articles varied from 2001 to 2017. Among them, eight were from Europe; two in Asia and one was conducted in Africa. The number of the patients in each study ranged from 40 [36] to 229 [26]. Five different *Lactobacillus* strains were used in these RCTs: (1) *L*. *gasseri* OLL2716 [26], (2) *L*. *casei* [27,28], (3) *L*. *acidophilus* [29], (4) *L*.*reuteri* [30–32,34, 36], (5) *Lactobacillus GG* [33, 35]. The duration of *Lactobacillus* supplementation ranged from 8 days [29] to 96 days [34]. Table 1 details the characteristics of each included study.

**Table 1:** Baseline characteristics of the included studies.

Study (year)	Location	Patients	Total cases (Exp/Cont)	*H*. *pylori* infection diagnosis (initial/rechecking)	Eradication regimen (dose/duration)	*Lactobacillus* regimen	Follow-up time	%Eradication (exp/cont) ITT	%Side effects (exp/cont)
Ryuzo D 2011	Japan (Asia)	Adults	229 (115/114)	RUT, Histology, Culture / ^13^C-UBT, HpSA	Rabeprazole 10 mg Bid amoxicillin 750 mg Bid clarithromycin 200 mg Bid, 7 days	*L*. *gasseri* OLL2716 (>10^9^CFU/g) 112 g bid, 4weeks	8 weeks	82.6/69.3	5.2/3.5
Sykora J 2005	Czech (Europe)	Children	86 (39/47)	Histology, HpSA, RUT / HpSA, UBT	omeprazole 20 mg/30kg Bid amoxicillin 25 mg/kg Bid clarithromycin 7.5mg/kg Bid, 7 days	*L*. *casei* (1x10^10^ CFU/day) 2 weeks	4 weeks	84.6/57.5	17.9/19.1
José E 2007	Spain (Europe)	Adults	71 (35/36)	Endoscopy, Histology, Serology /^13^C-UBT	omeprazole 20 mg Bid amoxicillin 1g Bid clarithromycin 500mg Bid, 7 days	*L*. *casei* (8 billion/day) tid, 4 weeks	8 weeks	94.0/76.0	NA
Silva M 2011	Portugal (Europe)	Adults	62 (31/31)	Culture, Histology/ ^13^C-UBT	omeprazole 20 mg Bid amoxicillin 1g Bid clarithromycin 500mg Bid, 8 days	*L*. *acidophilus* (2.5×10^10^ CFU/day) 8 days	6 weeks	83.9/80.6	NA
Veronica O 2012	Italy (Europe)	Adults	90 (45/45)	^13^C-UBT/ ^13^C-UBT	esomeprazole 20 mg Bid levofloxacin 500 mg Bid amoxicillin 1 g Bid, 7 days	*L*. *reuteri* (1x10^8^CFU/day) tid, 2 weeks	6 weeks	80.0/62.0	NA
Mohame H 2014	Egypt (Africa)	Adults	70 (35/35)	HpSA, RUT, Histology/ Histology	omeprazole 20 mg Bid amoxicillin 1g Bid clarithromycin 500mg Bid, 14 days	*L*. *reuteri* (2x10^8^ CFU/day) 4 weeks	4 weeks	74.3/65.7	28.5/68.5
Hania S 2009	Poland (Europe)	Children	66 (34/32)	RUT, Histology, ^13^C-UBT/ ^13^C-UBT	clarithromycin 10mg/kg Bid amoxicillin 25 mg/kg Bid omeprazole 0.5mg/kg Bid, 14 days	*Lactobacillus* GG (2x10^9^ CFU/day) 2 weeks	4–6 weeks	67.6/68.8	51.4/40.6
Shahraki T 2017	Iran (Asia)	Children	50 (25/25)	Histology/ ^13^C-UBT	clarithromycin 15mg/kg Bid amoxicillin 50mg/kg Bid omeprazole 1mg/kg Bid, 14 days	*L*. *reuteri* (1x10^9^ CFU/day) 4 weeks	4 weeks	88.0/76.0	NA
Francavilla R 2014	Italy (Europe)	Adults	88 (44/44)	RUT, Histology, Endoscopy/ 13C-UBT	omeprazole 20 mg bid, amoxicillin 1g bid, clarithromycin 500mg bid, 7 days	*L*. *reuteri* (2x108 CFU/day) 96 days	8 weeks	ITT:75.0/65.9	40.9/61.4
Armuzzi A 2001	Italy (Europe)	Adults	120 (60/60)	^13^C-UBT, IgG ELISA/ ^13^C-UBT, IgG ELISA	pantoprazole 40 mg bid, clarithromycin 500 mg bid, tinidazole 500 mg bid, 7 days	*Lactobacillus GG* (6x10^9^ CFU/day) 2 weeks	4 weeks	ITT:80.0/76.6	43.3/61.6
Lionetti E 2006	Italy (Europe)	Children	40 (20/20)	Histology, RUT/ ^13^C-UBT	omeprazole 1 mg/kg clarithromycin 15 mg/kg tinidazole 20 mg/kg Bid, 10 days	*L*.*reuteri* (1x10^8^CFU/day) 20 days	8 weeks	ITT:85.0/80.0	NA

*Bid* twice daily; *CFU* colony-forming unit; *HpSA H*. *pylori* stool antigen test; *NA* not available; *RUT* rapid urease test; *Tid* Three times daily; ^*13*^*C-UBT* ^13^C-urea breath test; *ITT* intent- to-treat *Exp/cont* experiment/control; *IgG ELISA IgG* enzyme linked immunosorbent assay

### Eradication rates

**Fig 2:**
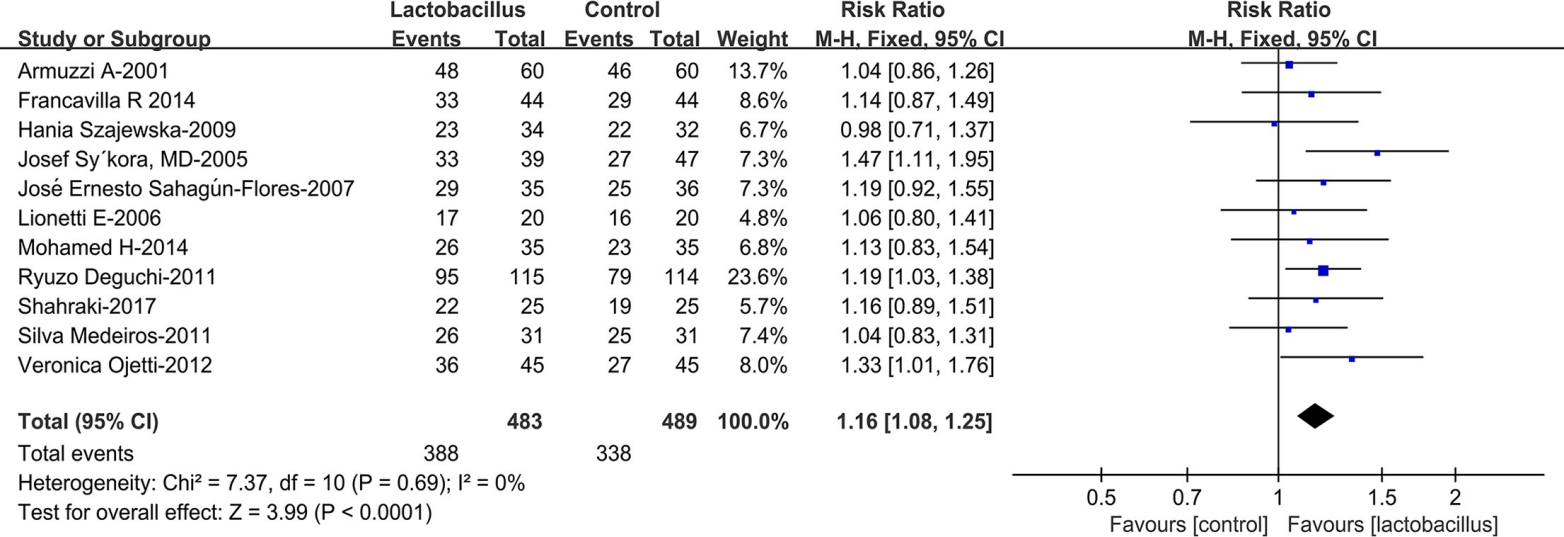
Eradication rates with or without *Lactobacillus* supplementation.

Among the involved cases, 483 patients were in the experimental group, 489 patients in the control group (Fig 2). The Mantel-Haensze fixed-effects model was used because of the low heterogeneity (*I*^2^ = 0%). The eradication rates in the *Lactobacillus* supplementation group were 80.3%, while 69.1% in the control group. The pooled RR was 1.16 (95% CI 1.08–1.25, *P*<0.0001), as showed via intention-to-treat analysis.

### Subgroup analysis

**Fig 3:**
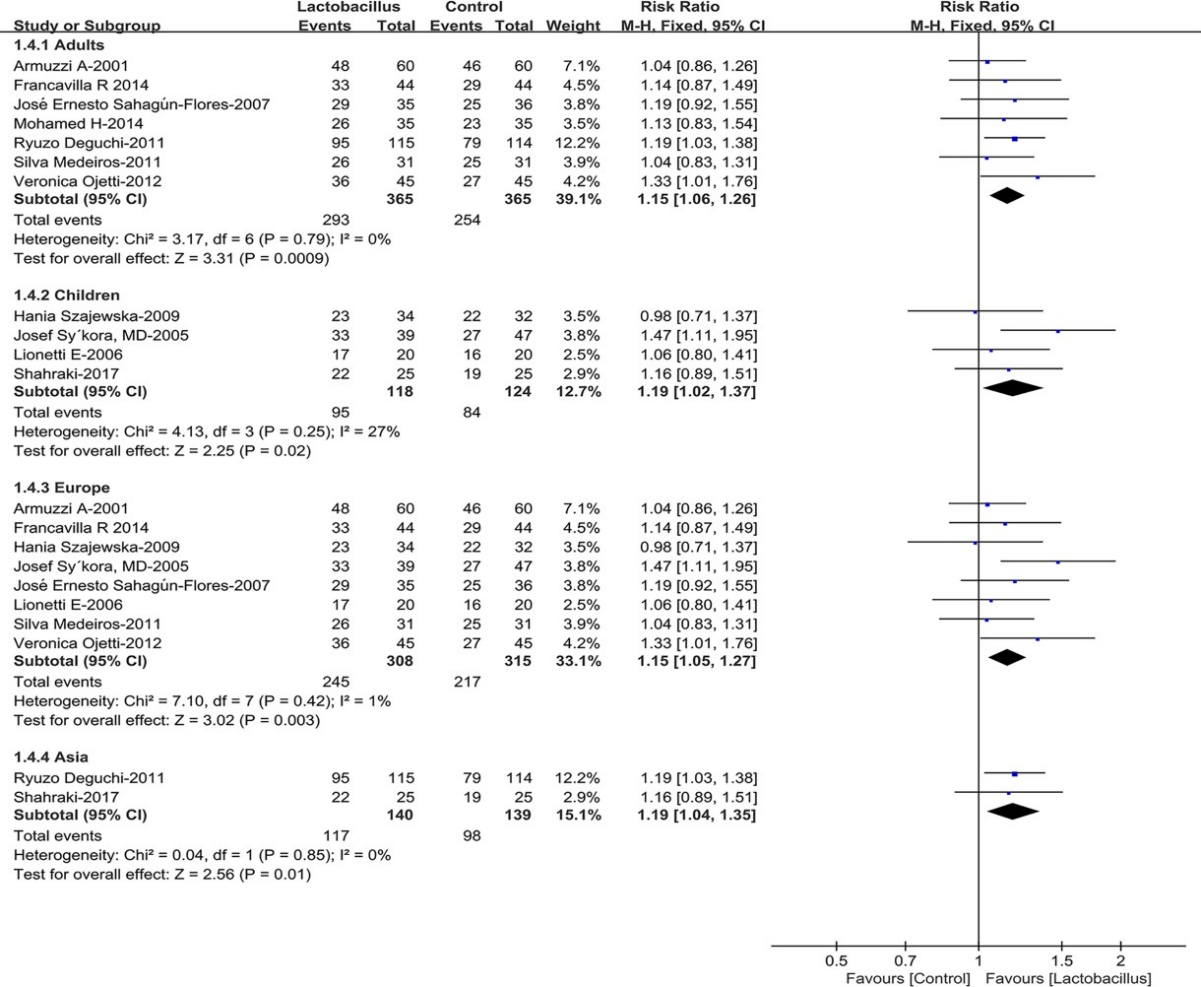
Eradication rates according to age and region.

**Fig 4:**
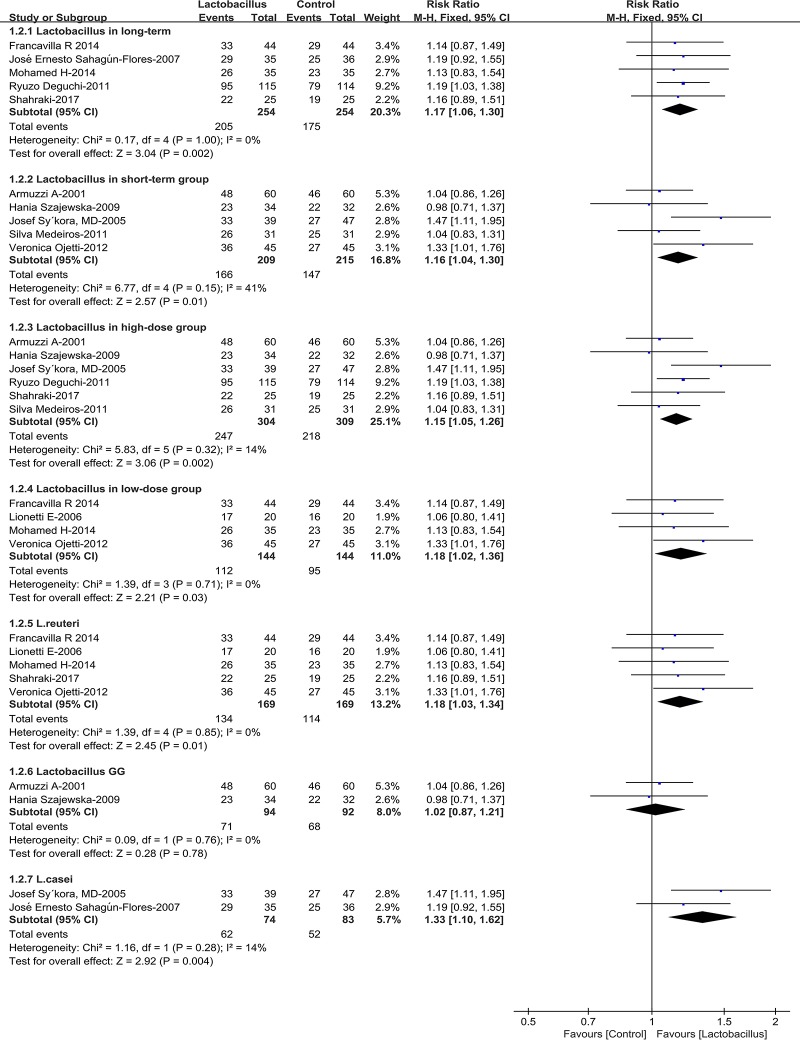
Eradication rates according to *Lactobacillus* supplementation duration, dose and species.

Subgroup analyses were conducted in our meta-analysis based on the following aspects regarding the outcomes of eradication rates: age of the patients (children or adults), location (Asia or Europe) (Fig 3), *Lactobacillus* supplementation duration (≤2 weeks, or ≥4 weeks), *Lactobacillus* species (*L*. *casei*, *L*. *reuteri or Lactobacillus GG*) and the dose of *Lactobacillus* supplementation (≤10^9^ CFU/day or ≥10^9^ CFU/day) (Fig 4). The subgroup analyses of age showed that the eradication rates in both adults and children subgroup increased significantly (adults pooled RR = 1.15, 95% CI 1.06–1.26, *P* = 0.0009; children RR = 1.19 95% CI 1.02–1.37, *P* = 0.02). Moreover, the pooled RRs in the Asia and Europe subgroups were 1.19 (95% CI 1.04–1.35, *P* = 0.01) and 1.15 (95% CI 1.05–1.27, *P* = 0.003), respectively, indicating that Asia and Europe subgroups had improved the eradication rates. Sub-analysis about *Lactobacillus* supplementation duration indicated that the pooled RR was 1.16 (95% CI 1.04–1.30, *P* = 0.01) in short-term subgroup, and 1.17 (95%CI 1.06–1.30, *P* = 0.002) in the long-term group. As for the dose of *Lactobacillus* supplementation (≤109 CFU/day or ≥109 CFU/day), subgroup analysis indicated that both high-dose group (RR = 1.15, 95% CI: 1.05–1.26, *P* = 0.002) and low-dose group (RR = 1.18, 95% CI: 1.02–1.36, *P* = 0.03) improved the *H*. *pylori* eradication rates. Interestingly, in subgroup analysis based on *Lactobacillus* species (*L*. *casei*, *L*. *reuteri* or *Lactobacillus GG*), the eradication rates increased significantly in the *L*. *reuteri* group and *L*. *casei* group (RR = 1.18, 95% CI:1.03–1.34, *P* = 0.01; RR = 1.33, 95% CI: 1.10–1.62, *P* = 0.004, respectively), nevertheless it seemed that *Lactobacillus GG* couldn’t improve the eradication rates (RR = 1.02, 95% CI: 0.87–1.21, *P* = 0.78). Other two *Lactobacillus* strains were not included in the *Lactobacillus* species subgroup analyses since the relevant data were only from a single trial [26, 29]. No significant heterogeneity was found in sub-group analysis.

### Side effects

**Fig 5:**
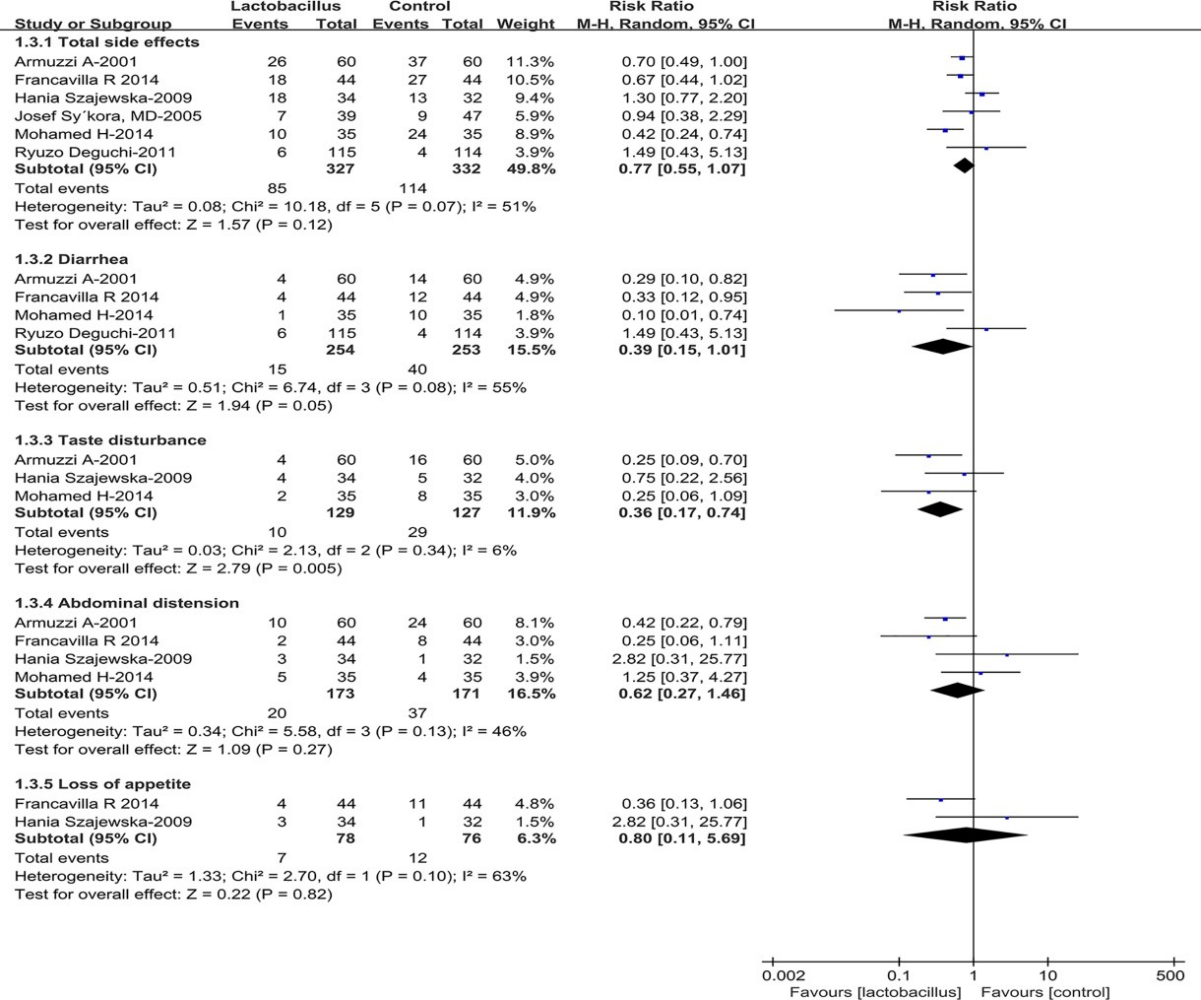
Effect of *Lactobacillus* supplementation on incidences of the side effects.

Six RCTs including 659 patients provided the side effect rates [26, 27, 31, 33–35]. The total side effect rates in the *Lactobacillus* supplementation group was 25.9%, while 34.3% in the control group. There was no significant difference between these two groups (RR = 0.77, 95% CI 0.55–1.07, *P* = 0.12) (Fig 5). Occurrence of certain specific symptoms resultant from the side effects, such as diarrhea, taste disturbance, loss of appetite and abdominal distension were also analyzed, respectively, in this study (Fig 5). *Lactobacillus* supplementation significantly reduced the incidence of taste disturbance (RR = 0.36, 95% CI 0.17–0.74, *P* = 0.005). However, there was no significant decrease in the incidence of diarrhea (RR = 0.39, 95% CI 0.15–1.01, *P* = 0.05), abdominal distension (RR = 0.62, 95% CI 0.27–1.46, *P* = 0.27) or loss of appetite (RR = 0.80, 95% CI 0.11–5.69, *P* = 0.82) in the experimental group, compared with the control group. Other individual side effects, such as nausea, constipation etc. were not analyzed here due to the limitation of the data obtained.

### Sensitivity analyses and publication bias

**Fig 6:**
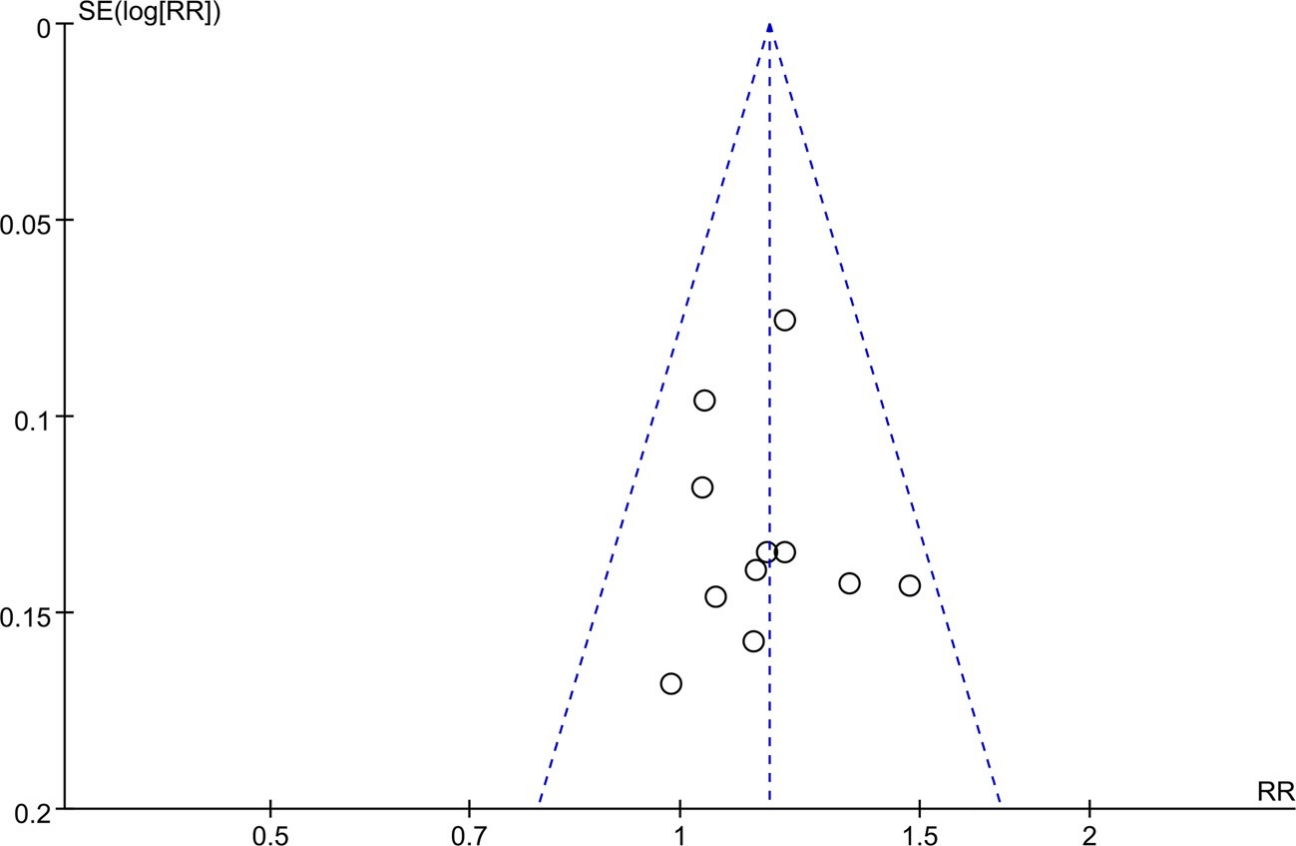
Funnel plot of the eradication rates of the included studies.

By removing one study at a time, none of the studies changed the pooled risk of the *H*. *pylori* eradication rates substantially, indicating the results of this study were reliable. The funnel plot obtained by an intentional analysis of the eradication rates revealed a slightly asymmetrical distribution (Fig 6). Nevertheless, no significant publication bias was detected by the Begg’s test (*P* = 1.000) and the Egger’s test (*P* = 0.931).

### Risk assessment of bias in the studies

**Fig 7:**
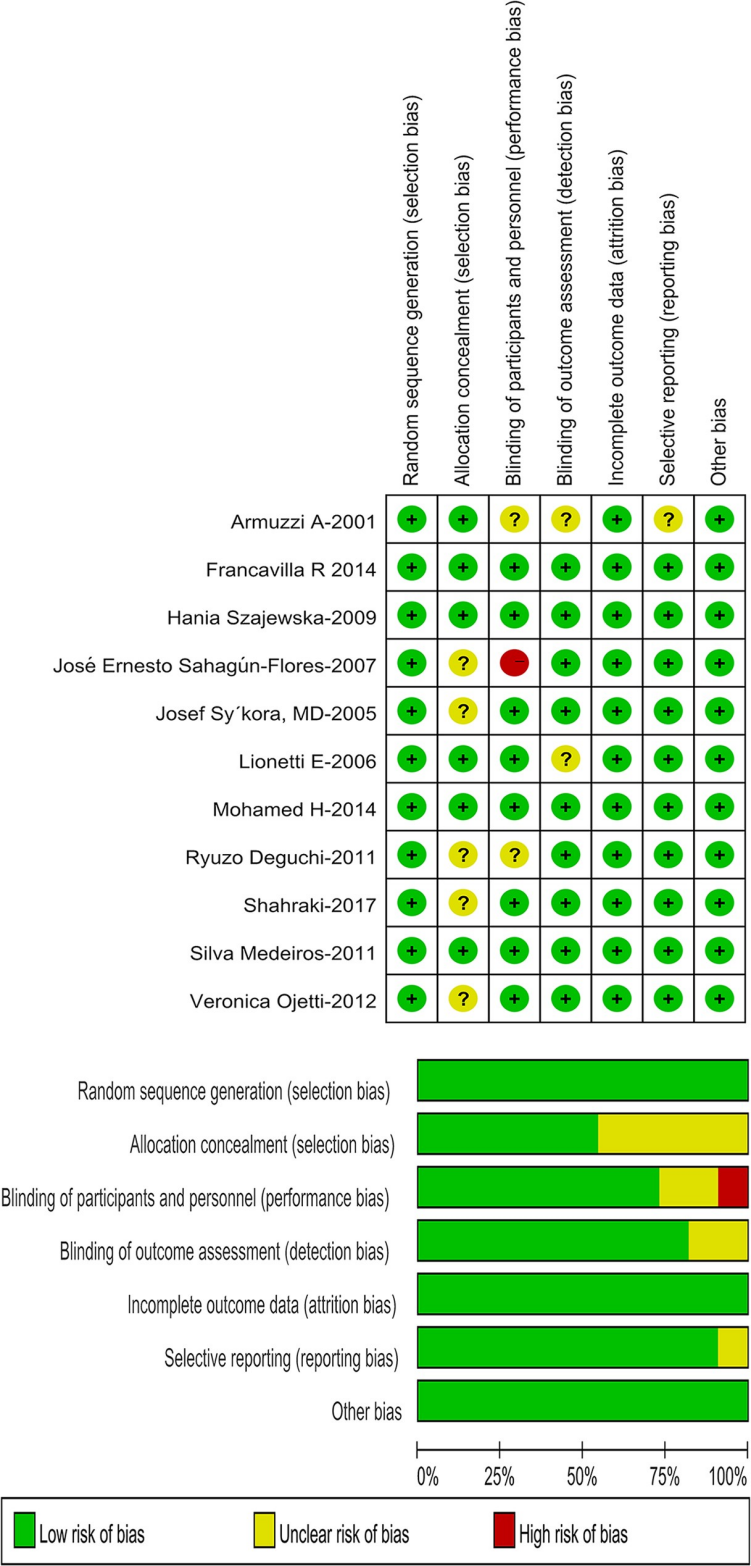
Risk of bias.

All the included studies had adequate and clear random sequence generation and blinding methods. One study [28] was judged as high risk of blinding of participants and personnel because the trial outcomes could be affected by the lack of blinding methods. Fig 7 shows the whole risk of bias summary. As for the quality of evidences, the reports on that the eradication rates, as the primary outcome, were enhanced by using *Lactobacillus* combined with traditional triple therapy eradication were graded as being of moderate quality, and evaluated as critical. Meanwhile, *Lactobacillus* supplementation reducing the chances of side effects as the second outcome was also graded as moderate, and evaluated as important. Table 2 details the GRADE quality of findings.

**Table 2:** GRADE quality of eradication regimens with or without *Lactobacillus*.

Outcomes	Illustrative comparative risks* (95% CI)	Relative effect (95% CI)	No. of participants (studies)	Quality of the evidence (GRADE)	Importance
	Assumed risk Corresponding risk				
	Control *Lactobacillus*				
Eradication rates	Study population	RR 1.16 (1.08 to 1.25)	972 (11 studies)	㊉ ㊉ ㊉ ㊀	
	691 per 1000 802 per 1,000 (747 to 864)			moderate	Critical
	Moderate				
	693 per 1000 804 per 1,000 (748 to 866)				
Side effects	Study population	RR 0.77 (0.55 to 1.07)	659 (6 studies)	㊉ ㊉ ㊉ ㊀	
	343 per 1000 264 per 1,000 (189 to 367)			moderate	Important
	Moderate				
	510 per 1000 393 per 1,000 (281 to 546)				

## Discussion

### Summary of evidence

In this meta-analysis, the eradication rates of *H*. *pylori* infection increased by 8.4% after the addition of *Lactobacillus* (pooled RR = 1.16, *P*<0.0001), which indicated *Lactobacillus* supplementation could significantly improve the eradication efficacy. Furthermore, in the subgroup analysis, the eradication rates in the *Lactobacillus* supplementation group increased by more than ten percent in both adult/children group and Asia/Europe group. In addition, the eradication rates of the *Lactobacillus* supplementation increased in both long-term/short-term groups and the high-dose/low-dose groups. With respect to *Lactobacillus* species, *Lactobacillus GG* couldn't improve the eradication rates, *L*. *casei* and *L*. *reuteri* seemed to be more effective in *H*. *pylori* eradication therapy. As for the adverse events, *Lactobacillus* supplementation could significantly reduce the risk of taste disturbance. However, the incidences of the total side effects might not decrease significantly. All the included RCTs featured clear and adequate randomization methods, and their results remained stable. All evidence for eradication rates and side effects was graded as moderate quality in our study according to the GRADE assessment. The results provide certain clinical implications for future researches. Because of the misuse or overuse of antibiotics in *H*. *pylori* eradication therapy, the antibiotics kill not only *H*. *pylori*, but also the normal flora, which could lead to the bacterial superinfection and *H*. *pylori* infection recurrence. At this point, the *Lactobacillus* supplementation might be helpful to reconstruct the micro-ecological environment.

### Experiments supporting the findings

Early studies showed that *Lactobacillus* strains could inhibit the attachment of *H*. *pylori* to gastric cell lines *in vitro* [37]. It has also been demonstrated in animal experiments that *Lactobacillus* could alleviate gastritis in mice infected with *H*. *pylori* [18]. At the same time, Sung et al. (2014) found the anti-*H*. *pylori* activity of the *Lactobacillus* strains isolated from baikkimchi [38]. Francavilla et al. found that *L*. *reuteri* could secrete reuterin, which has the anti-*H*. *pylori* effect [39]. Another study showed that *L*. *reuteri* could interfere with the binding of *H*. *pylori* to epithelial cell receptors through producing cell-surface proteins [40]. In addition, studies evidenced that *Lactobacillus GG* can inhibit the cell membrane leakage induced by *Helicobacter pylori* through improving the epithelial barrier [41]. Moreover, *Lactobacillus* might play a role in regulating the immune response. *L*. *acidophilus* could mitigate gastritis associated with *H*. *pylori* infection by inhibiting NFκB pathways [15], and *Lactobacillus GG* could also in *vitro* reduce the release of IL-8 induced by *H*. *pylori* [42]. Meanwhile, *Lactobacillus acidophilus* can also activate opioid and cannabinoid receptors, thus alleviating the discomfort caused by antibiotics and possibly reducing side effects [43].

### Strengths and limitations

The present study used the well-defined probiotics (*Lactobacillu*s), which has been confirmed effective for inhibiting *H*. *pylori* in clinical trials, to investigate the association of the bacteria of the same genus with the therapeutic efficacy. Besides, the RCTs included in our meta-analysis covered adults and children from Asia, Europe and Africa. Moreover, the Cochrane risk of bias assessment tool was used for quality assessment of the included studies, and the GRADE system was applied to evaluate the evidences of the key outcomes. The design of this study could improve the reliability of the conclusions.

However, this meta-analysis also has some limitations. First, the study included only 11 RCTs, which might influence the credibility of the outcomes. Next, the variety of population characteristics and the different duration of *Lactobacillus* supplementation may lead to clinical heterogeneity. Although subgroup analysis was conducted for breaking through this limitation, its influence may still be incompletely controlled. In addition, not all the studies included the side effect rates, and the severity of the adverse events was not evaluated here since relevant data was not mentioned in the included studies.

### Comparison with other studies

Our results proved *Lactobacillus* supplementation could significantly improve *H*. *pylori* eradication rate of the triple therapy, which showed accordance with some previous studies [44–46]. Feng et al.(2017) [47] identified that *L*. *casei* was more appropriate to supplement triple therapy for improving *H*. *pylori* eradication compared with other probiotic regimens, which was consistent with our findings. A study by Cindoruk et al. also showed the addition of *Lactobacillus GG* did not improve the eradication rate [48]. Besides, several probiotics, including *Saccharomyces boulardii* and *Bifidobacterium*, have also been used in combination with traditional therapies for eradication of *H*. *pylori* infection. Compared to standard triple therapies, although addition of *S*. *boulardii* reduced the incidence of diarrhea, it did not significantly improve the *H*. *pylori* eradication rate [49]. In another study, addition of the combination of *Lactobacillus acidophilus* and *Bifidobacterium* also failed to show an improvement in *H*. *pylori* infection eradication [50]. Subgroup analysis based on dose of *Lactobacillus* supplementation suggested that both low and high dose could enhance the efficacy of *H*. *pylori* eradication, which was consistent with Zheng et al. [44]. However, Fang et al. [46] drew inconsistent conclusions. As for the therapy-related adverse events, our results revealed *Lactobacillus* supplementation did not significantly reduce the incidence of total side effects, which was in accordance with Fang et al.[46] and Zheng et al.[44]. Furthermore, our results identified that *Lactobacillus* supplementation could reduce the incidence of taste disturbance, which was inconsistent with Lv et al. [51] and Li et al. [52]. A possible explanation for inconsistencies with other studies might be that the age distribution of the patients involved in the study was significantly different, and the duration and species of supplementation with *Lactobacillus* or probiotics might also be an important factor. Considering these factors, well-designed trials are needed to get more credible evidences.

### Conclusion

In conclusion, **c**urrent evidences suggest that *Lactobacillus* supplementation during treatment with standard triple therapy for *H*. *pylori* infection can significantly improve the eradication rates, and reduce the incidence of the taste disturbance. Future studies should focus on clarifying the optimal duration of *Lactobacillus* administration and an effective way to reduce the adverse events for enhancing the curative efficacy of the antibiotic-based therapies for *H*. *pylori* infection.

## Supporting information

S1 Table The complete search strategy. (DOC)

S2 Table PRISMA Check-list for this meta-analysis. (DOC)
