# Supplementary material for: Efficacy of Lactobacillus-supplemented triple therapy for H. pylori eradication: A meta-analysis of randomized controlled trials
Source: PLoS One. 2019 Oct 2;14(10):e0223309. doi: 10.1371/journal.pone.0223309 (PMC6774518; doi:10.1371/journal.pone.0223309)
Supplement: S1 Table — (DOC) [file pone.0223309.s001.doc]

**Table S1. Systematic literature review search terms and strategy**

| **Search terms for PubMed** |
| --- |
| #1 ("*Helicobacter pylori*"[Mesh] OR “*H. pylori*” [Title/Abstract] OR “*Campylobacter pylori*” [Title/Abstract] OR “*C. pylori*” [Title/Abstract]) |
| #2 (“*Lactobacillus”* [Mesh] OR “probiotics” [Title/Abstract] OR “probiotic” [Title/Abstract] OR “yogurt” [Title/Abstract] OR “yeast” [Title/Abstract]) |
| #3 (“randomized controlled trail”[Publication Type] OR “randomized” [Title/abstract] OR “placebo” [Title/abstract] OR “controlled clinical trials” [Publication Type] OR clinical trials [Publication Type]) |
| #1 AND #2 AND #3 |
| **Search terms for Embase** |
| #1 *Helicobacter pylori*/ OR *H. Pylori*. mp. OR *Campylobacter pylori*.mp. OR *C. pylori*/mp. |
| #2 *Lactobacillus*. mp. OR probiotics/ OR probiotic. mp. OR yogurt/ OR yeast.mp. |
| #3 randomized controlled trail .mp. OR controlled clinical trials / OR clinical trials .mp. OR randomized/ OR placebo.mp. |
| #1 AND #2 AND #3 |
| **Search terms for Web of Science** |
| TS=((“probiotics” OR “probiotic” OR “yogurt” OR “Lactobacillus” OR “yeast”) AND (“*Helicobacter pylori*” OR “*Campylobacter pylori*” OR “*H. pylori*” OR “*C. pylori*”) AND (“randomized controlled trail” OR “controlled clinical trials” OR “clinical trials” OR “randomized” OR “placebo”)) |
| **Search terms for Cochrane Library** |
| #1 MeSH descriptor: [*Helicobacter pylori*] explode all trees OR (*H. Pylori*):ti,ab,kw OR (*Campylobacter pylori*):ti,ab,kw OR (*C. Pylori*):ti,ab,kw |
| #2 MeSH descriptor: [*Lactobacillus*] explode all trees OR (probiotic):ti,ab,kw OR (probiotics):ti,ab,kw OR (yogurt):ti,ab,kw OR (yeast):ti,ab,kw |
| #3 #1 AND #2 |
